# Nanopore long-read sequencing for the critically ill facilitates ultrarapid diagnostics and urgent clinical decision making

**DOI:** 10.1038/s41431-025-01959-x

**Published:** 2025-10-20

**Authors:** Daphne J. Smits, Federico Ferraro, Mark Drost, Herma C. van der Linde, Bianca M. de Graaf, Yolande van Bever, Alice S. Brooks, Livija Bardina, Hennie T. Brüggenwirth, Christophe Debuy, Laura Donker Kaat, Bastiaan T. van Dijk, Nienke van Engelen, Geert Geeven, Raoul van de Graaf, Désirée Y. van Haaften-Visser, Peter M. van Hasselt, Daphne Heijsman, Yvonne M. C. Hendriks, Rebekkah J. Hitti-Malin, Lies H. Hoefsloot, Glenn Huijbregts, Hanna IJspeert, Sander Lamballais, Jona Mijalkovic, Merel O. Mol, Diënna Nawawi, Nadine Nederpelt, Esther A. R. Nibbeling, Wouter te Rijdt, Rachel Schot, Marjon van Slegtenhorst, Frank Sleutels, Eva L. M. Ulenkate, Monique Van Veghel – Plandsoen, Judith M. A. Verhagen, David Vos, Erwin Wauters, Martina Wilke, Marc Sylva, Tahsin Stefan Barakat, Tjakko J. van Ham, Tjitske Kleefstra, Dmitrijs Rots, Virginie J. M. Verhoeven

**Affiliations:** 1 Department of Clinical Genetics, Erasmus MC, University Medical Center Rotterdam, Rotterdam, the Netherlands; 2 Department of Paediatrics, Willem-Alexander Children’s Hospital, Division of Neonatology, Leiden University Medical Centre, Leiden, the Netherlands; 3 Department of Pediatrics, Center for Lysosomal and Metabolic Diseases, Erasmus MC, University Medical Center, Rotterdam, the Netherlands; 4 Department of Metabolic Diseases, Division Pediatrics, Wilhelmina Children’s Hospital University Medical Centre Utrecht, Utrecht University, Utrecht, the Netherlands; 5 Department of Clinical Genetics, Leiden University Medical Center, Leiden, the Netherlands; 6 Department of Neonatal and Pediatric Intensive Care, Erasmus MC-Sophia Children’s Hospital, University Medical Center Rotterdam, Rotterdam, the Netherlands; 7 Department Human Genetics, Radboud University Medical Center, Nijmegen, the Netherlands; 8 Center for Neuropsychiatry, Vincent van Gogh, Venray, the Netherlands

**Keywords:** Genetic testing, Genetic testing

## Abstract

Critically ill pediatric patients often have genetic disorders requiring a rapid diagnosis to guide urgent care decisions. Standard genetic testing typically takes weeks and requires multiple tests. Nanopore long-read genome sequencing (LR-GS) delivers genome-wide results within days as a one-test-fits-all solution. As one of the first centers in Europe, we implement ultrarapid LR-GS for critically ill patients. We enrolled 26 critically ill patients (median age 2 months) suspected of having a genetic disorder at the intensive care unit to perform (ultra)rapid nanopore LR-GS alongside standard genomic care. We compared diagnostic yield, turnaround time (TAT), and evaluated the impact on clinical decision making. In 11/26 cases a genetic diagnosis was made with (ultra)rapid LR-GS. From sample receipt to result, the average TAT was 5.3 days (range 2.0–10.8) for LR-GS and 18.4 days (range 6.1–29.1) for standard genomic care. DNA methylation analysis from LR-GS expedited the diagnosis in 3/26 cases. In 7/11 solved cases ultrarapid LR-GS led to immediate adjustments in patient care, e.g., medication switch or termination of treatment. Our findings underscore the clinical impact of ultrarapid LR-GS, including added value of methylation analysis, for critically ill patients and highlight existing challenges, paving the way to ultrarapid LR-GS integration into standard diagnostics.

## Introduction

Critically ill patients, specifically children, often have a high likelihood of having an underlying genetic disorder requiring a rapid diagnosis. Rapid (<10 days) and ultrarapid ( <3-5 days) genomic testing holds the potential to guide clinical management, offer treatment options, enhance prognosis, shorten irreversible suffering, shorten intensive care stays, reduce costs in critically ill patients, and reduce parental distress [1–4]. Standard genomic care, while comprehensive, has turnaround times (TAT) of several weeks and typically requires multiple tests. Current critical care demands swift decisions within a couple of days, so the results from genetic testing often cannot be readily integrated into treatment decision-making, delaying optimal care.

While various genome-wide approaches, such as short tandem repeat testing, short-read exome sequencing (ES) and genome sequencing (SR-GS), have been employed in rapid testing, they rely on short-read sequencing and do not capture DNA methylation (DNAm), requiring multiple tests for genetic and epigenetic disorders[1, 2]. Recent advancements in nanopore long-read genome sequencing (LR-GS) have enhanced sequencing quality, enabling ultrarapid genomic testing without the need for running samples in batches. Real-time native DNA LR-GS has the ability to detect and phase not only single nucleotide variants, but also structural variants, DNA methylation, repeat expansions, and variations in mitochondrial DNA. Therefore, LR-GS delivers comprehensive, clinically relevant insights—making it especially effective in time-sensitive clinical scenarios[5–8].

In this study, we assess the clinical utility of ultrarapid LR-GS sequencing for critically ill (pediatric) patients suspected of having a genetic disorder at the intensive care unit, comparing LR-GS to standard rapid genomic care, marking the first integration of ultrarapid LR-GS into standard diagnostics. The cases presented highlight the potential of ultrarapid LR-GS to reduce the number of tests needed, while enhancing clinical care and improving outcomes for patients with genetic conditions.

## Methods

### Participant inclusion

Individuals were included between May 2024 and May 2025. Individuals were eligible if they were critically ill, admitted to the intensive care unit (ICU) at Erasmus MC (Rotterdam, the Netherlands) or another hospital in the Netherlands, had a suspected genetic condition, and for whom standard rapid genomic care was requested at the clinical genetics laboratory at Erasmus MC. Individuals with a clear non-genetic etiology, or a previously confirmed genetic diagnosis, were excluded.

### DNA extraction and sequencing

High-quality genomic DNA was extracted from blood using either the ReliaPrep Large Volume HT gDNA Isolation kit (Promega, Leiden, The Netherlands) for larger blood volumes (>1 ml) or the Chemagic DNA Blood kit (Revvity, Baeswiler, Germany) for smaller blood volumes (<1 ml) by following the manufacturer’s instructions. DNA extraction followed standard methods used for standard diagnostics rather than preparations for ultrahigh molecular weight DNA. Following DNA fragmentation and short fragment removal, the sequencing library was prepared using the Oxford Nanopore Technology (ONT, Oxford, United Kingdom) ligation Sequencing Kit SQK-LSK114 according to the manufacturer’s instructions. For each individual, the library was loaded on two PromethION Flow cells R10.4.1 and sequenced to obtain read depth of 30-40x (runtime ~24 h) with read length targeting N50 ~ 15 kb. Samples were run as singletons or in trios, depending on the availability of parental DNA and/or a strong suspicion of a clinical diagnosis at the start of the isolation process.

### Data analysis

Basecalling with modification, and alignment to the reference genome (GRCh38) were performed live in MinKNOW v.24.02.19-v.24.11.11, using the SUP v.4.3.0 mode. DNA methylation was summarized with modkit v0.3.0. Variants were called using Clair3 [9], sniffles2 [10], SVision-pro [11] and Spectre (https://github.com/fritzsedlazeck/Spectre) and reads were phased using LongPhase v1.0.7 [12]. Variant annotation and Human Phenotype Ontology (HPO)-based prioritization was performed with Exomiser [13] including parental data when available. Exomiser annotations included REVEL [14], CADD [15], AlphaMissense [16] and SpliceAI [17] in silico tools for variant prioritization. Reported variants in the top 50 prioritized genes or combined score >0.75 were reviewed by both laboratory specialists and clinicians in relation to the patients’ presentation. Additional genes were analyzed, if requested by the referring clinical geneticist. Reported variants were validated by standard genomic care. Variants of interest were visualized using Integrative Genomics Viewer (IGV) before reporting. For variants of interest with low quality, targeted re-basecalling of loci of interest was performed using the more accurate and compute-intensive basecalling model SUP v5.0.0 using dorado (https://github.com/nanoporetech/dorado).

### DNA methylation signature analysis

As the available DNAm signature analysis is based on Illumina EPIC arrays, the ONT-derived DNA methylation calls were processed with an in-house script (https://github.com/f-ferraro/PseudoEpic-bedmethyl) to extract the beta values of the Illumina EPIC array sites, creating “PseudoEPIC” file. The beta values were submitted to EpigenCentral [18] for cases 1-9 and MethaDory [19] for cases 9-26, which allows for DNA methylation-based sample classification of 18 and 89 disorders, respectively.

### Clinical utility analysis

To further explore the impact of nanopore LR-GS results on clinical decision making, we applied the Clinician-reported Genetic testing Utility InDEx (C-GUIDE, version 1.2a) [20]. For each patient included in this study, the C-GUIDE questionnaire was completed retrospectively by the two members of the team (V.J.M.V. and D.J.S.) who had been involved in the diagnostic trajectory and disclosure of results for all cases. This approach ensured consistency in scoring and allowed for a clinically informed assessment of utility across the full cohort. Ratings included all relevant modules (primary variants and secondary variants) reflecting the perceived impact on care. Pharmacogenomic findings were not reported in this cohort and were, therefore, not scored. C-GUIDE scores were calculated by summing completed items across relevant sections (primary, secondary). Items not applicable (e.g., absent secondary findings) were excluded from scoring. C-GUIDE scores were interpreted contextually, with higher scores reflecting a greater number of clinician-reported impacts across domains such as diagnostic insight, management decisions, and psychosocial considerations.

## Results

### Diagnostic implementation of an (ultra)rapid Long-Read Genome Sequencing pipeline

**Fig. 1:**
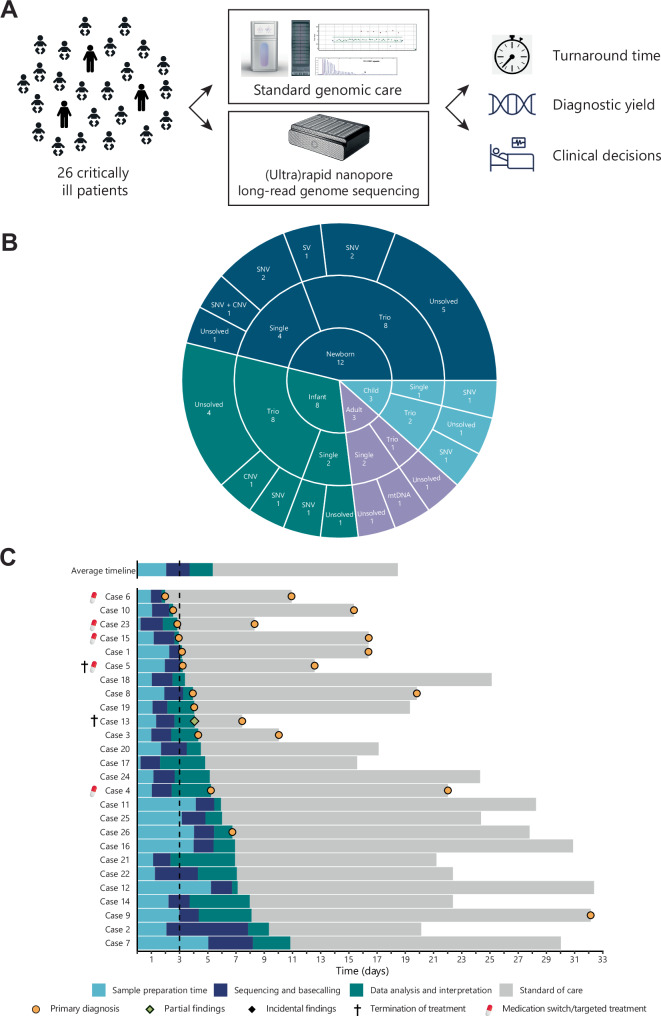
Study outline and cohort characteristics. **A** Outline of this study. **B** Sunburst chart summarizing the age group breakdown of the cohort analyzed, the analysis type and the classes of variants identified. **C** Comparison of the turnaround times for nanopore LR-GS and standard of care genetic testing, and overview of results impact on clinical decision making.

Patients were offered ultrarapid LR-GS after consultation at the ICU by a clinical geneticist. Following blood draw from both proband and parents (if available), both ultrarapid LR-GS and standard genomic care were initiated in parallel. We compared turnaround time, diagnostic yield and effect on clinical decision making after standard genomic care was finished (Fig. 1A).

Twenty six critically ill patients (23 children and three young adults) at the ICU of Erasmus MC were offered (ultra)rapid nanopore LR-GS. The median age at inclusion was 2 months, ranging from 3 days to 31.4 years. The final cohort consisted of 15 males and 11 females.

Using LR-GS, we obtained a genetic diagnosis for 11 out of 26 participants (42%; Fig. 1B). The average time from sample(s) receipt to result was 5.3 days (range 2.0-10.8 Fig. 1C) for LR-GS and 18.4 days (range 6.1-29.1) for standard genomic care. Ultrarapid LR-GS was significantly faster than standard genomic care (p = 4.01e-09, Mann-Whitney test). For LR-GS, the average sample preparation time was 2.0 days (range 5.7 h–5.2 days), the average sequencing time was 1.6 days (range 18.5 h–5.8 days) and the average data analysis and interpretation time was 1.6 days (range 30 min–4.67days). Ultrarapid LR-GS is a single test, while standard genomic care averaged 1.9 tests requested per patient (range 1–4).

### Ultrarapid long-read genome sequencing identifies diverse DNA variant types

**Fig. 2:**
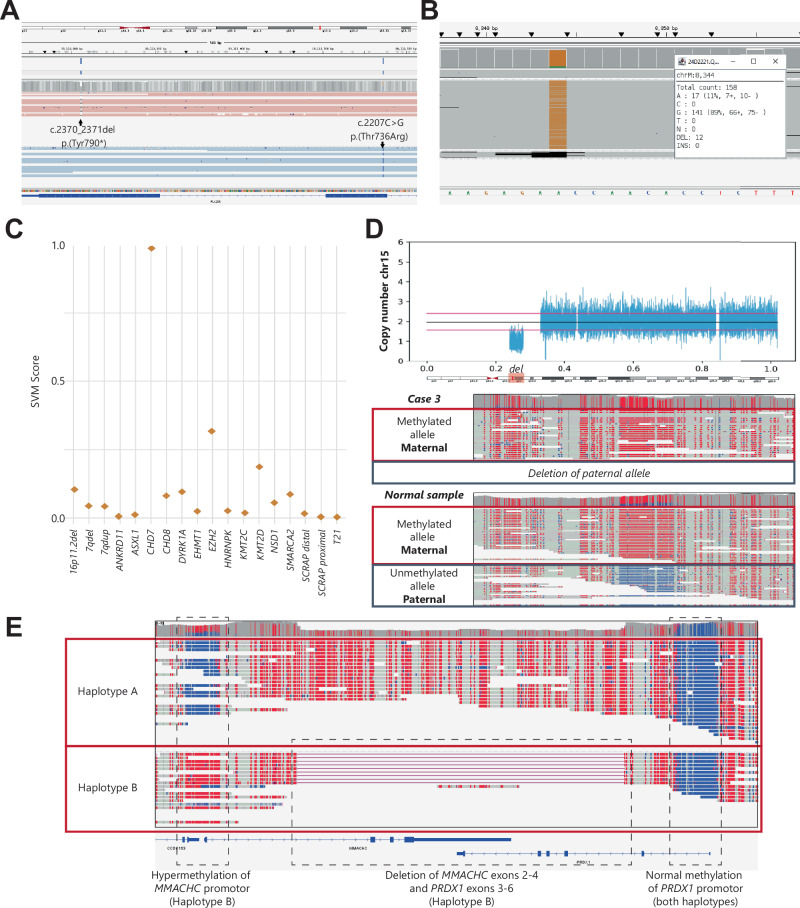
Diagnostic insights from nanopore long-read genome sequencing. **A** Case 10 with two *PLA2G6* pathogenic variants in different haplotypes (shown with blue and pink) confirming compound heterozygous inheritance. **B** Case 4 showing heteroplasmic (89%) pathogenic variant m.8344A>G; **C** Case 1 being classified as a CHARGE syndrome case on the *CHD7* DNAm signature (SVM > 0.5), confirming CHARGE syndrome molecularly, while being classified as control on the other tested signatures from EpigenCentral; **D** Case 3 showing a 15q11.2q13.1 deletion with loss of the unmethylated (paternal) allele, confirming Prader-Willi syndrome. Methylated cytosines are depicted in red, unmethylated in blue. Del: Deletion; **E** Case 23 showing hypermethylation of the *MMACHC* promoter on haplotype B, likely due to transcriptional readthrough starting from the *PRDX1* promotor (on the same haplotype) as a consequence of a ~ 14Kb deletion.

The primary molecular findings included various variant types, including SNVs/indels (heterozygous, compound heterozygous, homozygous, as well as heteroplasmic) and CNVs (Table 1**/**Fig. 1B). Importantly, LR-GS provided additional information (Fig. 2) for increasing diagnostic yield in a single test and/or reducing TATs. For example, LR-GS enabled direct phasing of compound heterozygous pathogenic variants in *PLA2G6* from the singleton patient data, confirming the autosomal recessive *PLA2G6*-associated neurodegeneration diagnosis (case 10; MIM#610217; Fig. 2A). Further, in case 4, we identified a pathogenic heteroplasmic (70%) mitochondrial DNA variant (m.8344A>G), leading to a diagnosis of MERRF syndrome (MIM#545000; Fig. 2B). For two cases, DNAm contributed to an early diagnosis. Case 1 was classified as CHARGE syndrome based on the DNA methylation signature, before genetic variants were called by the pipeline (Fig. 2C). This allowed for a targeted analysis of *CHD7*, which led to the identification of a *CHD7* frameshift variant within 48 h and confirmed the diagnosis of CHARGE syndrome (MIM#214800). Also, in case 3, a 15q11.2q13 deletion was found, for which methylation information enabled direct inspection of the known imprinted locus and recognition of a paternal allele deletion before parental sequencing data was available, confirming Prader-Willi syndrome diagnosis (MIM#176270; Fig. 2D). Additionally, we identified silencing of one of the *MMACHC* alleles in case 23. This is likely a secondary epivariant due to readthrough with the downstream gene [19]. While the partial gene deletion is diagnostic in this case, this showcases that LR-GS can also identify pathogenic epivariants, which is a common mechanism for several genes, including *MMACHC* [19] (Fig. 2E).

**Table 1:** Patient characteristics and diagnostic findings for standard care vs. (ultra)rapid long-read genome sequencing.

Case	Age	Analysis	Sex	Clinical presentation (HPO-terms)	Standard of care (number of molecular tests)	Variant; zygosity, inheritance mode, ACMG classification [34, 35]	Detected by		Aberration type	Final diagnosis, inheritance (OMIM)	Impact on clinical decision making, C-GUIDE clinical utility index score^$^
							Standard care	LR-GS			
1	N	Single	M	Choanal atresia, optic disc coloboma, ectopic kidney	ES, SNP-array (2)	*CHD7* variant*; heterozygous, de novo, pathogenic ACMG: PVS1 + PS2 + PM2	√	√	SNV	CHARGE syndrome, AD (#214800)	C-GUIDE score: 20
						CHARGE DNAm signature	X	√	DNAm		
2	I	Single	M	Cardiac arrest	ES (1)	No diagnosis	NA	NA	NA	NA	C-GUIDE score: 2
3	I	Trio	F	Respiratory insufficiency, intrauterine growth retardation, neonatal hypotonia	ES, MS-MLPA, *DMPK, SMN1* (4)	seq[GRCh38] del(15)(q11.2q13.1) NC_000015.9: g.(23327281_23502849)_(28134728_28322732)del; heterozygous, de novo, pathogenic^#^ ACMG: 2 A + 4 L Type II deletion chr15q11.2q13.1 region on paternal allele	√	√	CNV	Prader-Willi syndrome, AD (#176270)	C-GUIDE score: 21
						Hypermethylation on chr15q11.2q13.1	X	√	DNAm		
4	A	Single	F	Seizures, respiratory insufficiency, cerebellar atrophy, abnormal brain morphology, increased circulating lactate concentration, muscle weakness, ataxia	ES, mtDNA (2)	NC_012920.1(*MT-TK*):m.8344A>G; 89% heteroplasmy, pathogenic^#^ ACMG: PS4_Very strong + PP1_mod + PS3_Sup + PP3	√	√	mtDNA	Myoclonic epilepsy associated with ragged-red fibers (MERFF), MT (#545000)	Avoidance of mitochondrial-toxic drugs C-GUIDE score: 23
5	N	Trio	M	Myoclonic spasms, epileptic encephalopathy	ES (1)	NM_172107.3(*KCNQ2*):c.601C>T, p.(Arg201Cys); heterozygous, de novo, pathogenic^#^ ACMG: PS2 + PM1 + PM2 + PM5 + PP3	√	√	SNV	Developmental and epileptic encephalopathy 7, AD (#121200)	C-GUIDE score: 16 Vigabatrin with no specific effect, followed by termination of treatment
6	C	Single	M	Seizure, failure to thrive, deep white matter hypodensities, premature graying of hair, hearing impairment	ES, SNP-array (2)	NM_183236.3(*RAB27A*):c.514_518del, p.(Gln172Asnfs*2); homozygous, pathogenic^#^ ACMG: PVS1_Strong + PM2 + PM3_Strong	√	√	SNV	Griscelli syndrome type 2, AR (#607624)	C-GUIDE score: 23 Dexamethasone, intrathecal prednisolone, methotrexate for hemophagocytic lymphohistiocytosis, eligibility for HSCT
7	N	Single	M	Central apnea	ES, SNP-array. *PHOX2B* (3)	No diagnosis	NA	NA	NA	NA	C-GUIDE score: 0
8	N	Single	M	Esophageal atresia, horseshoe kidney, anuria	ES, SNP-array (2)	NM_002480.3(*PPP1R12A*):c.2157dup, p.(Arg720Glnfs*6); heterozygous, de novo, pathogenic^$^ ACMG: PVS1 + PS2 + PM2	√	√	SNV	Genitourinary and/or/brain malformation syndrome, AD (#618820)	C-GUIDE score: 15
9	I	Trio	F	Intrauterine growth retardation, thrombocytopenia, increased laxity of fingers, long fingers, long toe, premature birth, downslanted palpebral fissures, failure to thrive	SNP-array (p), ES (p), SR-GS (3)	NM_017654.4(*SAMD9*):c.3846C>A, p.(Asn1282Lys); heterozygous, de novo, likely pathogenic^$^ ACMG: PS2 + PM2 For LR-GS: Called in secondary analysis but not prioritized by Exomiser, likely due to benign in silico predictions	√	X	SNV	MIRAGE syndrome, AD (#617053)	C-GUIDE score: 0
10	I	Single	F	Fasciculations, hypotonia, generalized hypotonia, motor regression, developmental regression	ES, *SMN1* (2)	NM_003560.4(*PLA2G6*):c.2370_2371del, p.(Tyr790*); heterozygous, paternal, pathogenic^#^ ACMG: PVS1 + PM2 + PM3_Strong + PP5	√	√	SNV	*PLA2G6*-associated neurodegeneration, AR (#610217)	C-GUIDE score: 18
						NM_003560.4(*PLA2G6*):c.2207C>G, p.(Thr736Arg); heterozygous, maternal, likely pathogenic^$^ ACMG: PM1_Sup + PM2 + PM3 + PP3	√	√	SNV		
						NM_024496.4(*IRF2BPL*):c.1263dup, p.(Glu422*); heterozygous, de novo, pathogenic^$^ ACMG: PVS1 + PS2 + PM2 For LR-GS: bug in Exomiser pipeline. Second finding.	√	X	SNV	Neurodevelopmental disorder with regression, abnormal movements, loss of speech, and seizures, AD (#618088)	
11	N	Trio	F	Bilateral vocal cord paresis, small for gestational age, periventricular white matter hyperintensities	SR-GS (1)	No diagnosis	NA	NA	NA	NA	C-GUIDE score: 0
12	I	Trio	M	Dilated cardiomyopathy, congestive heart failure	SR-GS, mtDNA (2)	No diagnosis	NA	NA	NA	NA	C-GUIDE score: 1
13	N	Trio	M	Congenital malformation of the right heart, esophageal atresia, cryptorchidism, abnormal heart morphology, total anomalous pulmonary venous return, small for gestational age	ES (p) (1)	NM_004247.4(*EFTUD2*):c.349G>C, p.(Asp117His); heterozygous, de novo, variant of uncertain significance^$^ ACMG: PS2 + PM2 (sufficient for likely pathogenic, yet classified as variant of uncertain significance because of *EFTUD2* intragenic deletion in cis). SVision-pro added to variant calling pipeline following this case.	√	√	SNV	Mandibulofacial dysostosis, Guion-Almeida type, AD (#603892)	C-GUIDE score: 16 Termination of treatment
						seq[GRCh38] del(17)(q21.31) chr17:g.44867345_44881820del heterozygous, de novo, likely pathogenic^$^ ACMG: 2E Deletion ( ~14Kb) of exon 7-13 of the *EFTUD2* gene For LR-GS: CNV not called by variant caller (sniffles2) in secondary analysis	√	X	CNV		
14	N	Trio	M	Pulmonary hypoplasia, pulmonary arterial hypertension, respiratory insufficiency, neonatal asphyxia, pneumothorax, bell-shaped thorax	SR-GS, *FOXF1, SMN1* (3)	NM_001139441.1(*BCAP31*):c.436_439dup, p.(Ala147Glyfs*10); heterozygous, maternal, likely pathogenic^$^ ACMG: PVS1 + PM2 For LR-GS: Not prioritized because of HPO terms mismatch. Incidental finding.	√	X	SNV	Deafness, dystonia, and cerebral hypomyelination, XLR (#300475)	C-GUIDE score: 6
15	C	Trio	M	Severe global developmental delay, developmental regression, motor regression, loss of speech, impaired oropharyngeal swallow response, hypotonia, symmetric lesions of the basal ganglia, focal T2 hyperintense basal ganglia lesion, focal T2 hyperintense thalamic lesion, focal T2 hyperintense brainstem lesion	SR-GS, mtDNA (2)	NM_025243.4(*SLC19A3*):c.952G>A, p.(Ala318Thr), homozygous, likely pathogenic^#^ ACMG: PM1 + PP5 + PP4 + PM2	√	√	SNV	Biotin-thiamine-responsive basal ganglia disease; AR (#607483)	C-GUIDE score: 23 Biotin + thiamine
16	I	Trio	F	Dilated cardiomyopathy, congestive heart failure	SR-GS, mtDNA (2)	No diagnosis	NA	NA	NA	NA	C-GUIDE score: 0
17	N	Trio	F	Hypertrophic cardiomyopathy, recurrent infantile hypoglycemia, muscular ventricular septal defect, atrial septal defect	SNP-array (p), ES (p), SR-GS, mtDNA (4)	No diagnosis	NA	NA	NA	NA	C-GUIDE score: 2
18	A	Trio	M	Super-refractory status epilepticus, abnormal thalamic MRI signal intensity, EEG with burst suppression, seizure, bilateral tonic-clonic seizure with focal onset, abnormal thalamic MRI signal intensity, seizure, bilateral tonic-clonic seizure with focal onset, hypoglutaminemia	,SR-GS(1)	No diagnosis	NA	NA	NA	NA	C-GUIDE score: 0
19	N	Trio	F	Abnormal mitral valve physiology, abnormal tricuspid valve physiology, abnormal left ventricular function, abnormal right ventricular function, left ventricular hypertrophy, ventricular septal defect	SR-GS, mtDNA (2)	No diagnosis NM_001492.6(*GDF1*):c.681C>A, p.(Cys227*), heterozygous, maternal, pathogenic^#^ ACMG: PVS1, PS4, PM2 Incidental finding.	√	√	NA	NA	C-GUIDE score: 5
20	A	Single	M	Cardiomyopathy, low-output congestive heart failure, myocarditis	SR-GS, mtDNA (2)	No diagnosis	NA	NA	NA	NA	C-GUIDE score: 6
21	I	Trio	M	Cerebral ischemia, cerebral infarct, status epilepticus	SR-GS, mtDNA (2)	No diagnosis	NA	NA	NA	NA	C-GUIDE score: 0
22	N	Trio	M	Partial agenesis of the corpus callosum, retrognathia, splenic cyst, abnormality of the respiratory system, impaired oropharyngeal swallow response, seizure	SR-GS mtDNA (2)	No diagnosis	NA	NA	NA	NA	C-GUIDE score: -1
23	N	Single	M	Methylmalonic aciduria (NBS), methylmalonic acidemia (NBS), atrial septal defect, thin corpus callosum	SR-GS (1)	NM_015506.3(*MMACHC*):c.271dup, p.(Arg91Lysfs*14), paternal, pathogenic^#^ ACMG: PVS1, PM3, PM2	√	√	SNV	Methylmalonic aciduria and homocystinuria, cblC type; AR (#277400)	C-GUIDE score: 23 Targeted treatment for cobalamin C deficiency
						seq[GRCh38] del(1)(p34.1) chr1:g.45504427_45518399del, maternal, pathogenic^$^ ACMG: 2E (likely pathogenic, yet classified as pathogenic because in trans of pathogenic SNV) Deletion ( ~14Kb) of exon 2-4 of the *MMACHC* gene and exon 3-6 of the *PRDX1* gene	√	√	CNV		
						Hypermethylation of MMACHC promotor on deleted allele	X	√	DNAm		
24	C	Trio	F	Thromboembolic stroke, ischemic stroke	SR-GS (1)	No diagnosis	NA	NA	NA	NA	C-GUIDE score: 0
25	N	Trio	F	Pancytopenia, increased circulating ferritin concentration, elevated circulating hepatic transaminase concentration, histiocytosis, hemophagocytosis	SR-GS (1)	NM_182918.4(*ERG*):c.1162dup, p.(Tyr388Leufs*78), paternal, variant of uncertain significance^$^ ACMG: PVS1, PM2 (sufficient for likely pathogenic, yet classified as variant of uncertain significance because of lack of OMIM gene-phenotype).	X	√	SNV	ERG-related bone marrow failure and hematological malignancy [22]; AD (no OMIM number)	C-GUIDE score: 3 (Patient died before results came in)
26	I	Trio	F	Seizure, epileptic spasm, developmental regression, depressed nasal bridge, epicanthus inversus, hypertelorism, global developmental delay	SR-GS (1)	NM_000548.5(*TSC2*):c.3284+1G>A, p.(?), heterozygous, de novo, pathogenic^#^ ACMG: PVS1_Strong, PS2, PM2	√	√	SNV	Tuberous sclerosis-2; AD (#613254)	C-GUIDE score: 21

Overview of all cases, in order of sample arrival, the HPO terms and the molecular findings identified in the analysis (abbreviations: LR-GS: long-read genome sequencing, *N* newborn ( ≤ 28 days), *I* infant ( < 1 year), *C* child, *A* adult, *M* male, *F* female, *NA* not applicable, *ES* Exome Sequencing, *SR-GS* short-read genome sequencing, *NBS* newborn screening, (*p*) prenatal, *SNV* single nucleotide variant, *CNV* copy number variant, *DNAm* DNA methylation, *mtDNA* mitochondrial DNA, *DMPK* *DMPK* gene repeat expansion testing with RP-PCR, *SMN1*: *SMN1* deletion testing with MLPA*,* *AD* autosomal dominant, *AR* autosomal recessive, *XLR* X-linked recessive), *MT* mitochondrial inheritance. Variant annotation is based on hg38. *Patient/parents did not provide consent for full disclosure of the specific DNA variant. ^#^ Recurrent variant (variant described in ClinVar or HGMD database), ^$^ Novel variant (variant not described in ClinVar or HGMD database); ^$^ C-GUIDE scores reflect cumulative clinician-reported utility; higher scores indicate greater impact. Range: -1.0–23.0 (all), 15.0–23.0 (genetically solved cases with LR-GS).

A challenge we faced during manual curation of the variants in IGV was distinguishing true from false positive truncating variants in homopolymer repeats, given by the current error rate of the ONT technology in these regions. In these instances, manual targeted re-basecalling using an updated basecalling model (SUP v5.0.0), not yet included in the latest MinKNOW, was resolutive.

Importantly, we identified a number of differences between LR-GS and regular testing results. In two cases, pathogenic variants were missed by the LR-GS pipeline. In case 9, a pathogenic *SAMD9* variant was not prioritized by Exomiser likely due to benign in silico prediction scores (REVEL = 0.03; AlphaMissense = 0.463), highlighting limitations of AI-driven variant prioritization and the need of expert curation. In case 13, structural variant caller sniffles2 did not identify an *EFTUD2* ~14kb intragenic deletion even though it was clearly evident in the LR-GS read data, while an adjacent de novo *EFTUD2* missense variant, likely a product of the reparation process, was identified and prioritized [21]. As the *EFTUD2* missense variant was reported by LR-GS, the clinical diagnosis did not change after the results from standard genomic care. This case highlights the challenges of current tools to detect small CNVs (in the range of read N50). After adjusting the bioinformatic pipeline with additional tools, we were able to retrospectively detect the *EFTUD2* deletion, and could also identify an *MMACHC* deletion of similar size (case 23).

In contrast, due to poor genotype–phenotype matching of the gene description in OMIM, standard analysis software missed a VUS in *ERG* (Lymphatic Malformation 14, MIM #620602) in case 25, which was detected by LR-GS. The patient had suspected hemophagocytic lymphohistiocytosis, and recent reports link *ERG* variants to a bone marrow failure phenotype [22], possibly explaining the presentation better than the available OMIM entry. This results in a diagnostic yield of 46% (12/26) for standard genomic care. Additionally, in two cases (case 10 and 14), standard testing revealed a pathogenic variant that was interpreted as a second diagnosis or as an incidental finding with important implications for future management. In case 10, in addition to the compound heterozygous *PLA2G6* variants already reported after ultrarapid LR-GS, standard genomic care identified a heterozygous pathogenic variant in *IRF2BPL* (Neurodevelopmental disorder with regression, abnormal movements, loss of speech, and seizures; MIM#618088). This variant was considered a second diagnosis and had been missed by LR-GS due to an error in the Exomiser pipeline. In case 14, a hemizygous *BCAP31* variant was considered incidental, as the presenting features did not match the *BCAP31*-associated phenotype (Deafness, dystonia, and cerebral hypomyelination, MIM #300475) and did not explain the patient’s initial presentation. In total, in three cases (case 9, 14 and 25), differences in the results between LR-GS and standard genomic testing were due to the different platforms employed for variant prioritization and interpretation and in two cases (cases 10 and 13) – because of the technical limitations of the utilized LR-GS bioinformatic pipeline, which were resolved after the adjustment of the pipeline.

Fifteen of the cases remained without a genetic diagnosis, also following standard genomic care, including later data reanalysis prompted by novel symptoms. In two cases (case 19 and 25), variants were reported that require further follow-up. In case 19, a heterozygous pathogenic variant in *GDF1* (Congenital heart defects, multiple types 6, MIM #613854) was identified. The patient’s main phenotype was cardiomyopathy, which resolved with treatment, alongside a small ventricular septal defect in an otherwise normal heart. As *GDF1* is mainly associated with complex congenital heart defects, this variant was considered an incidental finding in this context. In case 25, a VUS in *ERG* was reported that could potentially be linked to the phenotype, as described in the preceding paragraph. In all other unsolved cases, no variants were prioritized or reported after comprehensive analysis. One unresolved case (case 7) presenting with central hypoventilation improved over time and is no longer suspected of having a genetic condition.

### Impact of ultrarapid long-read genome sequencing on clinical decision making

**Fig. 3:**
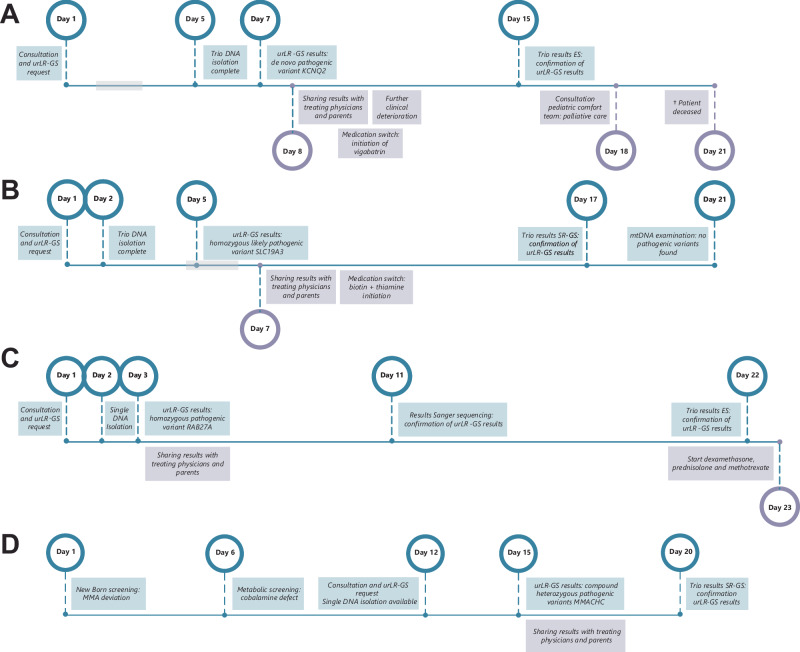
Timelines of diagnostic process and clinical impact of ultrarapid long-read genome sequencing. **A** Case 5 with *KCNQ2*-related Developmental and epileptic encephalopathy 7 (MIM#121200); **B** Case 15 with *SLC19A3*-related Biotin-thiamine-responsive basal ganglia disease (MIM#607483); **C** Case 6 with *RAB27A*-related Griscelli syndrome type 2 (MIM#607624); **D** Case 23 with Methylmalonic aciduria and homocystinuria, cblC type (MIM#277400). Grey blocks represent weekends; only shown when significant for impact/delay. urLR-GS ultrarapid long-read genome sequencing, ES Exome Sequencing, SR-GS short-read genome sequencing, MMA Methylmalonic acid.

Seven of the individuals with a genetic diagnosis identified via LR-GS (64%; Table 1**/**Fig. 3) received significant and immediate adjustments to their clinical management and/or therapeutic approaches. In three cases, a medication switch was implemented. In case 4 with MERRF syndrome (MIM#545000), mitochondrial-toxic drugs were avoided. In case 5 with *KCNQ2*-related Developmental and epileptic encephalopathy 7 (MIM#121200), vigabatrin was initiated based on literature-reported efficacy [23] seven days prior to standard genomic care results, but due to a lack of effect, treatment was discontinued (Fig. 3A). In case 15 with Biotin-thiamine-responsive basal ganglia disease (MIM#607483), biotin and thiamine therapy was initiated to prevent further deterioration ten days before diagnostic SR-GS confirmed the (ultra)rapid result [24] (Fig. 3B). In two cases, the diagnosis enabled targeted treatment options. In case 6 with Griscelli syndrome type 2 (MIM#607624) the diagnosis on day 3 led to initiation of dexamethasone, intrathecal prednisolone, and methotrexate for hemophagocytic lymphohistiocytosis, with subsequent eligibility for HSCT (Fig. 3C). In case 23 with cobalamin C deficiency (MIM#277400), the genetic diagnosis enabled targeted treatment, improved understanding of the phenotype, and informed the clinical prognosis. Notably, case 23 was admitted following abnormal findings in the national newborn screening program. This case illustrates that ultrarapid LR-GS can also play an important role in rapidly confirming abnormal newborn screening results, thereby supporting early diagnosis and clinical decision-making (Fig. 3D). In two cases (case 5 with *KCNQ2*-related Developmental and epileptic encephalopathy (MIM#121200) and case 13 with *EFTUD2*-related Mandibulofacial dysostosis, Guion-Almeida type (MIM#603892)), the diagnoses prompted discussions regarding termination of treatment, including the involvement of the pediatric comfort team due to poor prognosis [25].

To systematically evaluate the impact of LR-GS on clinical decision making, we applied the C-GUIDE instrument to each case (Table 1). Total C-GUIDE scores ranged from -1 to 23, with a median of 5.5 (IQR 19.5). Among the genetically solved cases (*n* = 11), total scores were consistently higher, with a median of 21.0 (IQR 6.0; range 15.0–23.0), reflecting that LR-GS contributed meaningfully to diagnostic resolution, informed clinical management, and supported patient-centered care.

## Discussion

This study demonstrates the feasibility and clinical utility of (ultra)rapid genetic testing with LR-GS, providing both DNA variant and methylation analysis, for diagnosing critically ill individuals. This approach improves clinical management and guides personalized care by significantly reducing TATs and enables the detection of diverse genetic and epigenetic variant types while reducing the number of tests needed per patient.

The use of ultrarapid genetic testing shortened the time to diagnosis by nearly two weeks and directly impacted clinical management in almost two thirds of cases with an identified genetic diagnosis. While shortening of the TAT for ~2 weeks has previously been shown to have significant impact on clinical outcome and costs-of-care, our ultimate goal would be to deliver a genetic diagnosis within 48–72 h [3, 26]. In four of the cases presented here, this goal was achieved (Fig. 1B). The initiation phase of our study highlighted several challenges towards consistently reaching this TAT, including blood sample logistics, technical setbacks (e.g., the need for additional DNA extraction because of the required amount of DNA for ultrarapid LR-GS on two flowcells), analytical complexities, access to equipment and limitations in available specialized personnel during out-of-office hours. Further streamlining of these aspects will contribute to reducing TAT to the aimed 48–72 h.

Most of the differences observed between (ultra)rapid LR-GS and regular testing could be attributed to the alternative variant prioritization and interpretation platforms employed, which will be resolved by the adoption of a single platform in the next stage of implementation. Furthermore, we identified limitations of the employed structural variant caller (case 13), also used by other (ultra)rapid LR-GS studies [7], which prompted the adoption of multiple tools for SV calling (e.g., SVision-pro). LR-GS bioinformatic tools are still improving and ongoing optimization of the utilized tools and the bioinformatic pipeline is necessary. Similarly, future improvements in the basecalling algorithms will decrease the number of false positive truncating variants in homopolymer repeats, reducing curation time and facilitating variant curation. Addressing these challenges will be essential to enhance yield and processing speeds and to be most impactful in urgent clinical settings. Moreover, follow-up studies will be required to evaluate the experiences from parents and caregivers, as well as the cost-effectiveness of this technique. While sequencing costs for are currently higher for LR-GS than SR-GS, both are currently reimbursed equally in the Netherlands, while ultrarapid LR-GS has broader economic impact, including reductions in additional clinical testing and ICU stay duration. Because this requires detailed multi-faceted evaluation, we are conducting a dedicated cost-effectiveness study which will comprehensively assess both direct and indirect healthcare costs associated with ultrarapid LR-GS implementation at ICU.

While recent studies showed the additional diagnostic yield after using LR-GS compared to short-read sequencing [27], all variant types identified in this cohort were also reported from standard care, but multiple assays were required (1.9 on average; Table 1). We additionally experienced the added value of LR-GS technology, including (genome-wide) methylation analysis, identification of a mitochondrial DNA variant, as well as the use of phasing; not only to increase diagnostic yield, but also to provide the final diagnosis quicker, without the need of consecutive testing (e.g., segregation of a recessive variant, identification of DNAm signature or confirming PWS in a case with the 15q11.2q13 deletion) [28]. Importantly, as the number of known DNAm signatures and pathogenic epigenetic variants continues to grow [29], the added value of DNAm detection from LR-GS is likely to become increasingly significant for rapid diagnosis.

Various genetic techniques, including targeted panels, ES, and SR-GS have been applied in rapid genomic testing initiatives [30–32]. Although SR-GS is also feasible within ultrarapid timeframe ( < 3–5 days) [26], it requires sample pooling, has limited capacity for variant phasing, detecting structural variants [33], and does not allow for methylation detection, all of which are possible with LR-GS. In published (ultra)rapid genomic testing studies, LR-GS approaches are scarcely examined. Gorzynski et al. demonstrated the feasibility of ultrarapid nanopore LR-GS for the first time in critically ill patients, achieving a diagnosis in 5 of 12 cases, with a shortest time-to-diagnosis of 7 h and direct clinical impact [5]. However, they utilized 48 R9 flowcells per sample, which is not achievable in diagnostic settings (cost-wise, or sample-volume-wise). Nyaga et al. utilized rapid nanopore LR-GS in an acute care setting in 14 trio samples in research lab settings, reaching a 5-workday average turnaround time, but did not provide absolute day count [6]. Kamolvisit et al. assessed the use of singleton rapid nanopore LR-GS as a first-tier genetic test in critically ill patients, diagnosing 61% of 18 enrolled cases with a median turnaround time of 9 days and treatment adjustments or procedural interventions implemented in 61% [8]. Our findings further confirm the utility of (ultra)rapid LR-GS, with the addition of DNA methylation, for critically ill patients in a clinical diagnostic setting. We have used a trio rather than singleton approach in the majority of the cases, while also achieving the fastest reported turnaround times for (ultra)rapid nanopore LR-GS. Notably, our approach incorporated methylation analysis, which contributed to expedited diagnoses in specific cases. We demonstrate a high diagnostic yield (42%) achieved with (ultra)rapid LR-GS, an average time-to-diagnosis of 5.9 calendar days from consultation, prompting changes in clinical decision making in 63.6% of the solved cases, as further supported by systematic C-GUIDE analyses showing high clinical utility scores.

In conclusion, this report demonstrates the road to the first implementation of (ultra)rapid nanopore LR-GS into standard genomic care for critically ill (pediatric) patients. Our findings demonstrate ultrarapid diagnostics provided by this single technology, which has an important impact on critical care management by enabling timely, informed clinical decisions that may significantly improve patient outcomes.

## Data Availability

All reported findings are provided in this manuscript. Genome sequencing data is not available due to the sensitive nature of the data.
